# Cooled Radiofrequency Ablation for Bilateral Greater Occipital Neuralgia

**DOI:** 10.1155/2014/257373

**Published:** 2014-02-06

**Authors:** Tiffany Vu, Akhil Chhatre

**Affiliations:** Department of Physical Medicine & Rehabilitation, School of Medicine, The Johns Hopkins University, 600 N. Wolfe Street, Baltimore, MD 21287, USA

## Abstract

This report describes a case of bilateral greater occipital neuralgia treated with cooled radiofrequency ablation. The case is considered in relation to a review of greater occipital neuralgia, continuous thermal and pulsed radiofrequency ablation, and current medical literature on cooled radiofrequency ablation. In this case, a 35-year-old female with a 2.5-year history of chronic suboccipital bilateral headaches, described as constant, burning, and pulsating pain that started at the suboccipital region and radiated into her vertex. She was diagnosed with bilateral greater occipital neuralgia. She underwent cooled radiofrequency ablation of bilateral greater occipital nerves with minimal side effects and 75% pain reduction. Cooled radiofrequency ablation of the greater occipital nerve in challenging cases is an alternative to pulsed and continuous RFA to alleviate pain with less side effects and potential for long-term efficacy.

## 1. Introduction

Greater occipital neuralgia (GON) is a challenging condition in physiatric, neurology, and pain management ambulatory clinics [1, 2]. A wide variety of treatments have been reported but no standard criterion exists [2]. Conservative treatments involve physical therapy, icing, avoiding compression of the nerve, nonsteroidal anti-inflammatory and nerve medications, manual decompression, and local occipital nerve blockade. If these measures fail, patients do have the option to pursue surgical care. Nonsurgical, minimally invasive options include phenol chemodenervation and radiofrequency ablation (RFA) of the affected nerve. Pulsed and continuous RFA for GON [2–4] has been described in current literature for greater occipital nerve ablation; however, cooled radiofrequency ablation has not. In this case report, we briefly review greater occipital neuralgia and compare the differences between pulsed, continuous, and cooled RFA in the treatment of this syndrome.

## 2. Case Presentation (Methods and Results)

A 35-year-old female presented to a physiatric outpatient clinic with a 2.5-year history of chronic suboccipital bilateral headaches that radiated to the posterior scalp and vertex of her head. The pain was described as constant, burning, and pulsating with a severity of 5/10 on the verbal analog scale (Numeric Pain Scale) but has intermittently increased to 10/10 in the previous past 2 weeks. Associated symptoms include insomnia, imbalance, muscle aches, nausea, neck pain, scalp tenderness, tingling, and numbness. The aggravating factors for the pain included flexion, extension, or rotation of neck position, fatigue, medications, weather changes, and bright light. Mitigating factors included ice packs and pain medication, specifically Fioricet provided mild relief. In the recent months leading up to the office visit, she was taking 4 Fioricet tablets in a day. Additionally, in the past she has tried physical therapy, chiropractic treatment, massage, acupuncture, NSAIDS, and several pain and nerve medications that provided mild relief. She had pulsed RFA, continuous RFA, and greater and lesser steroid and anesthetic occipital nerve blocks in the past. She reported that her experience with continuous RFA was extremely painful and did not provide relief to her pain. Her experience with pulsed RFA resulted in moderate pain relief of approximately 50% but returned to 100% after 2 weeks.

The past medical history includes systemic lupus erythematosus and three motor vehicle accidents with the last one in 2010. Her current medications included Fioricet, Plaquenil, levonorgestrel-ethinyl estradiol, and ondansetron.

Physical examination revealed exquisite tenderness of the suboccipital region upon palpation with radiation into the area over the vertex of the head. This is worsened with neck rotation in passive and active range of motion. The remainder of the examination was normal.

Review of recent flexion and extension cervical radiographs revealed no joint space or disk narrowing, and vertebral body height was normal. No acute pathology was demonstrated. No instability with flexion and extension. Report from magnetic resonance imaging revealed mild disk bulging at C5-C6 level slightly eccentric to the right and mild moderate right-sided neuroforaminal narrowing but otherwise normal.

Bilateral greater occipital nerve blockade under ultrasound guidance for needle positioning was done on initial evaluation using 0.2 mL of 2% lidocaine (preservative-free) mixed with 0.8 mL of dexamethasone solution (10 mg/mL). She responded positively to the GON blocks with 95% relief for five days. This blockade was diagnostic and therapeutic. We decided to pursue cooled radiofrequency ablation with hopes of significant pain relief and improvement in symptomatology.

Subsequently, 18 days later, she underwent cooled RFA of bilateral GON. The patient perceived sensory stimulation at 0.6 V. Cooled RFA was performed at 40°C for 150 seconds. Immediately after the procedure, she had 100% relief of pain. Seven hours after the procedure, she continued to have 100% relief of pain in the suboccipital and occipital regions but felt the similar presenting tingling, pulsating pain near the vertex of her head. There were no complications after the procedure. Prior to cooled RFA procedure, she took 4 Fioricet daily with only minimal improvement of pain. 24 hours later, she has experienced sustained relief with 75% reduction of pain after using 1 Fioricet and ice pack.

## 3. Discussion

Occipital neuralgia is defined as paroxysmal stabbing pain in the distribution of the greater, lesser, and third occipital nerves. It can be associated with diminished sensation or dysaesthesia and commonly has tenderness over the affected nerve that is eased temporarily by local anesthetic nerve blockade [5, 6]. The diagnosis is difficult to decipher and can be confounded by other headache causes that can respond to nerve blocks as well, such as cervicogenic, migraine, atlanto-axial or upper zygapophyseal joints pathology, and posttraumatic syndrome.

Occipital neuralgia can be caused by entrapment of the occipital nerves, spondylosis, idiopathic, head trauma, compression from extra- or intracranial vessels, giant cell arteritis, mass or tumor, and tendinomuscular compression. The majority of occipital neuralgia cases (85%) involve the greater occipital nerve. Localization of the greater occipital nerve can be challenging. Anatomically, the greater occipital nerve is comprised of the dorsal primary ramus of C2 and C3. It emerges with the lesser occipital nerve between the first and second cervical vertebrae, arises from the suboccipital triangle between the nuchal muscles, and then passes through the trapezius muscle to innervate the posterior scalp to the vertex [3, 6].

There is a lack of evidence supporting long-term benefit in pharmacotherapies, topical analgesics, and occipital nerve blocks [2]. Peripheral nerve stimulation is invasive and expensive but has been seen in small, uncontrolled studies to provide long-term benefit [7]. Radiofrequency ablation of the greater occipital nerves is a minimally invasive procedure that should be considered for challenging cases prior to invasive surgery.

Radiofrequency ablation was introduced in 1974 to treat various pain syndromes [8] and is the use of a radiofrequency lesion generator that heats tissue to a selected temperature making a distinct lesion at a target nerve. Traditional continuous radiofrequency ablation uses extremely high frequency alternating current to create coagulative necrosis at the precise target nerve. This is advantageous because it avoids lesioning of the wrong nerves. Generally, focal tissue destruction occurs between 60 and 80°C [9]. Unfortunately, this procedure has risks such as deafferentation pain syndrome [9], neuritis, and parenthesis [10, 11]. Additionally, limitations of continuous radiofrequency ablation were the limited size of the lesions and the depth was restricted within the tissue [12].

Pulsed radiofrequency ablation was developed as an alternative to continuous thermal RFA with the intended advantage of avoiding the complications of deafferentation pain syndrome [3]. It involves induction of a low intensity, short duration with high voltage over 60 radiofrequency pulses in the vicinity of the sensory nerves. Theoretically, the fibers responsible for conduction of nociceptors, myelinated A-delta, and small unmyelinated C fibers are dampened [2]. The maximum electrode temperature is 45°C which is below neurodestructive levels [2, 3]. Pulsed RFA has been shown to have relatively less unfavorable effects than continuous RFA, but the long-term efficacy of pulsed RFA has not been established [3, 10, 12].

Cooled radiofrequency ablation is a newer alternative to continuous and pulsed RFA. It has been used in cardiac electrophysiology [13] and tumor reduction [14]. Internally cooled radiofrequency probes operate as a heat sink by dissipating heat from tissue closest to the electrode, decreasing the chance of charring the tissue [15] which is a complication in continuous RFA. If the electrodes in continuous RFA were larger or the temperature increased, the tissue would char and self-limit the energy added and the size of the lesion. In contrast, the lesions from cooled RFA are deeper and spherical rather than elliptical and markedly larger in volume. This leads to a higher probability of successful neurodestruction of a pain generator [12]. Similar to continuous RFA, the size of the resultant lesion in cooled RFA depends on electrode temperature, probe size, duration of the treatment, and local tissue features. For example, using 50°C isotherm, an 18-gauge electrode is used with its tip heated to 55°C to 60°C for 150 seconds, the subsequent lesion from cooled RFA is approximately 8 to 10 mm in diameter [12, 16].

The usage of cooled RFA in greater occipital neuralgia has not been documented in the literature. However, there are few published articles on the treatment of sacroiliac and discogenic pain using cooled RFA. Ho et al. investigated cooled radiofrequency denervation for treatment of sacroiliac joint pain in 20 cases and it was found that 15 of the 20 patients showed a significant reduction in pain (at least 3-point reduction on the Numeric Rating Scale) at 2 years following cooled RFA [15]. Cohen et al. conducted a randomized placebo-controlled study evaluating lateral branch radiofrequency denervation for sacroiliac joint pain. 14 of the 28 participants in the study received L4-L5 primary dorsal rami and S1–S3 lateral branch radiofrequency denervation using cooling RFA after a local anesthetic block, and 14 patients received the local anesthetic block followed by placebo denervation. Patients who did not respond to placebo injections crossed over and were treated with radiofrequency denervation using conventional technology [17]. At 1-month comparison, cooled RFA showed significant lower pain and disability scores. Malik et al. compared the usage of cooled RFA to continuous RFA. The case revealed that the patient had minimal pain relief at 3- and 12-week follow-up after continuous RFA of the left L2 through L5 medial branches. At 6 months, the patient received cooled RFA in the same region. The patient reported significant pain relief with pain scores reduced to 2-3/10 on the Numeric Pain Scale [12]. More recently, Patel et al. published a randomized, placebo-controlled study to assess the efficacy of lateral branch neurotomy using cooled RFA for chronic sacroiliac joint pain. At 3-month follow-up, 47% of the 51 treated patients and 12% of sham subjects achieved significant improvements in quality of life and global perceived effect. At 6 and 9 months, respectively, 38% and 59% of treated subjects achieved treatment success [18].

In our case, the patient had trialed numerous other treatment options including several nerve blocks, physical therapy, adjuvant pain and nerve medications, and pulsed and continuous radiofrequency ablation. She could not tolerate continuous radiofrequency ablation and it was found to be minimally effective. Cooled RFA is an alternative that provided 75% relief in a very difficult to treat case of greater occipital neuralgia that is both tolerable and has potential for long-term efficacy. However, full effects cannot be determined without follow-up at 3, 6, and 12 months.

## 4. Conclusion

Our case represents the first report describing management of greater occipital neuralgia by usage of cooled radiofrequency ablation. Greater occipital neuralgia can be challenging to treat as there is no universal standard. Although the literature is limited in the usage and efficacy of cooled RFA, this treatment is an alternative option to pulsed and continuous RFA with a better safety profile and potential long-term efficacy. The thermal lesions ensuing after cooled RFA is significantly larger in volume and spherical in shape and can be made deeper; therefore, theoretically it has a higher probability in neurodestruction of the pain generators from the GON. Even so, there is a need for further scientific review with randomized, controlled trials for cooled radiofrequency ablation in the treatment of greater occipital neuralgia as well as other spinal pain syndromes.
